# Methyl 4-chloro-3,5-dinitro­benzoate

**DOI:** 10.1107/S1600536809051630

**Published:** 2009-12-04

**Authors:** Ya-Ling Liu, Pei Zou, Min-Hao Xie, Hao Wu, Yong-Jun He

**Affiliations:** a Jiangsu Institute of Nuclear Medicine, Wuxi 214063, People’s Republic of China

## Abstract

In the mol­ecule of the title compound, C_8_H_5_ClN_2_O_6_, the two nitro groups and the ester group make dihedral angles of 29.6 (1)°, 82.3 (1)° and 13.7 (1)°, respectively, with the benzene ring. In the crystal structure weak C—H⋯O inter­actions are present.

## Related literature

For the use of the title compound as a herbicide, see: Akira *et al.* (1978 ▶); Ferenc *et al.* (1984 ▶).
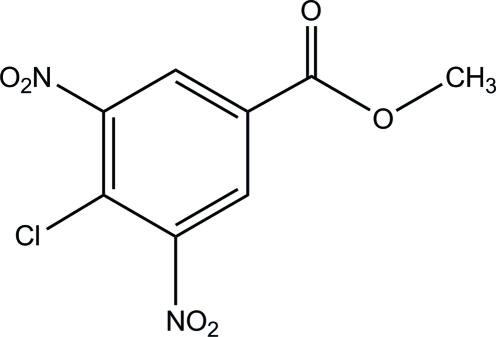


## Experimental

### 

#### Crystal data

C_8_H_5_ClN_2_O_6_
*M* *_r_* = 260.59
Triclinic, 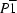

*a* = 4.8579 (10) Å
*b* = 9.4438 (19) Å
*c* = 11.369 (2) Å
α = 73.36 (3)°
β = 88.09 (3)°
γ = 87.47 (3)°
*V* = 499.14 (18) Å^3^
*Z* = 2
Mo *K*α radiation
μ = 0.40 mm^−1^
*T* = 93 K
0.50 × 0.33 × 0.17 mm

#### Data collection

Rigaku SPIDER diffractometer
Absorption correction: multi-scan (North *et al.*, 1968 ▶) *T*_min_ = 0.824, *T*_max_ = 0.936
3935 measured reflections
2193 independent reflections
1607 reflections with *I* > 2σ(*I*)
*R*_int_ = 0.029
Standard reflections: 0

#### Refinement

*R*[*F* ^2^ > 2σ(*F* ^2^)] = 0.047
*wR*(*F* ^2^) = 0.142
*S* = 1.02
2193 reflections
155 parameters
H-atom parameters constrained
Δρ_max_ = 0.85 e Å^−3^
Δρ_min_ = −0.32 e Å^−3^

### 

Data collection: *RAPID-AUTO* (Rigaku 2004 ▶); cell refinement: *RAPID-AUTO*; data reduction: *RAPID-AUTO*; program(s) used to solve structure: *SHELXS97* (Sheldrick, 2008 ▶); program(s) used to refine structure: *SHELXL97* (Sheldrick, 2008 ▶); molecular graphics: *ORTEP-3 for Windows* (Farrugia, 1997 ▶); software used to prepare material for publication: *SHELXL97*.

## Supplementary Material

Crystal structure: contains datablocks I, global. DOI: 10.1107/S1600536809051630/xu2697sup1.cif

Structure factors: contains datablocks I. DOI: 10.1107/S1600536809051630/xu2697Isup2.hkl

Additional supplementary materials:  crystallographic information; 3D view; checkCIF report

## Figures and Tables

**Table 1:** Hydrogen-bond geometry (Å, °)

*D*—H⋯*A*	*D*—H	H⋯*A*	*D*⋯*A*	*D*—H⋯*A*
C1—H1⋯O1^i^	0.95	2.51	3.329 (3)	145
C5—H5⋯O6^ii^	0.95	2.34	3.143 (3)	142

Symmetry codes: (i) 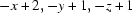; (ii) 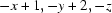.

**Fig. 1.**
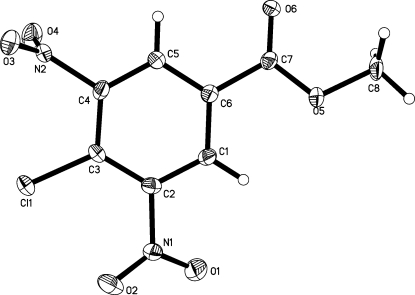
A view of the title compound with the atomic numbering scheme. Displacement ellipsoids were drawn at the 30% probability level.

## supplementary crystallographic information

### Comment

The title compound (Fig.1) is useful as a herbicide (Akira *et al.*,1978; Ferenc *et al.*, 1984). It give good result as seed-dressing fungicide for sunflower, corn and flax. We report here the crystal structure of the title compound. Two nitro groups (O1/N1/O2 and O3/N2/O4) attached at C2 and C4, the ester group(O5/C7/O6) attached at C6 form dihedral angles of 150.4 (1)°, 97.7 (1)° and 166.3 (1)° with the mean plane of the C1-benzene ring, respectively. In the crystal structure, adjacent molecules are linked through weak C—H···O hydrogen interactions (Table 1).

### Experimental

A sample of commercial methyl 4-chloro-3,5-dinitrobenzoate (Aldrich) was crystalized by slow evaporation of a solution in methanol: colourless chunk-shaped crystals were formed after several days.

### Refinement

H atoms are positioned geometrically, with C—H = 0.95 and 0.98 Å for benzene and methyl H atoms respectively, and are allowed to ride on the C atoms to which they are bonded, with *U*_iso_(H) = 1.5*U*_eq_(C) for methyl H atoms and *U*_iso_(H) = 1.2*U*_eq_(C) fot the aromatic H atoms.

### Crystal data

**Table:**

C_8_H_5_ClN_2_O_6_	*Z* = 2
*M**_r_* = 260.59	*F*(000) = 264
Triclinic, *P*1	*D*_x_ = 1.734 Mg m^−^^3^
Hall symbol: -P 1	Mo *K*α radiation, λ = 0.71073 Å
*a* = 4.8579 (10) Å	Cell parameters from 1306 reflections
*b* = 9.4438 (19) Å	θ = 3.7–27.5°
*c* = 11.369 (2) Å	µ = 0.40 mm^−^^1^
α = 73.36 (3)°	*T* = 93 K
β = 88.09 (3)°	Chunk, colorless
γ = 87.47 (3)°	0.50 × 0.33 × 0.17 mm
*V* = 499.14 (18) Å^3^	

### Data collection

**Table:**

Rigaku SPIDER diffractometer	2193 independent reflections
Radiation source: Rotating Anode	1607 reflections with *I* > 2σ(*I*)
graphite	*R*_int_ = 0.029
ω scans	θ_max_ = 27.5°, θ_min_ = 3.3°
Absorption correction: multi-scan (North *et al.*, 1968)	*h* = −6→6
*T*_min_ = 0.824, *T*_max_ = 0.936	*k* = −9→12
3935 measured reflections	*l* = −13→14

### Refinement

**Table:**

Refinement on *F*^2^	Primary atom site location: structure-invariant direct methods
Least-squares matrix: full	Secondary atom site location: difference Fourier map
*R*[*F*^2^ > 2σ(*F*^2^)] = 0.047	Hydrogen site location: inferred from neighbouring sites
*wR*(*F*^2^) = 0.142	H-atom parameters constrained
*S* = 1.02	*w* = 1/[σ^2^(*F*_o_^2^) + (0.0772*P*)^2^] where *P* = (*F*_o_^2^ + 2*F*_c_^2^)/3
2193 reflections	(Δ/σ)_max_ = 0.001
155 parameters	Δρ_max_ = 0.85 e Å^−^^3^
0 restraints	Δρ_min_ = −0.32 e Å^−^^3^

### Special details

**Table:**

Geometry. All e.s.d.'s (except the e.s.d. in the dihedral angle between two l.s. planes) are estimated using the full covariance matrix. The cell e.s.d.'s are taken into account individually in the estimation of e.s.d.'s in distances, angles and torsion angles; correlations between e.s.d.'s in cell parameters are only used when they are defined by crystal symmetry. An approximate (isotropic) treatment of cell e.s.d.'s is used for estimating e.s.d.'s involving l.s. planes.
Refinement. Refinement of *F*^2^ against ALL reflections. The weighted *R*-factor *wR* and goodness of fit *S* are based on *F*^2^, conventional *R*-factors *R* are based on *F*, with *F* set to zero for negative *F*^2^. The threshold expression of *F*^2^ > σ(*F*^2^) is used only for calculating *R*-factors(gt) *etc*. and is not relevant to the choice of reflections for refinement. *R*-factors based on *F*^2^ are statistically about twice as large as those based on *F*, and *R*- factors based on ALL data will be even larger.

### Fractional atomic coordinates and isotropic or equivalent isotropic displacement parameters (Å^2^)

**Table:**

	*x*	*y*	*z*	*U*_iso_*/*U*_eq_	
Cl1	1.27729 (13)	1.05900 (7)	0.37558 (6)	0.0216 (2)	
O1	0.9997 (5)	0.6244 (2)	0.56066 (17)	0.0317 (5)	
O2	1.3643 (4)	0.7546 (2)	0.51374 (19)	0.0345 (5)	
O3	1.1854 (4)	1.2028 (2)	0.07808 (17)	0.0249 (5)	
O4	0.8170 (4)	1.2738 (2)	0.16400 (18)	0.0278 (5)	
O5	0.4877 (4)	0.58546 (19)	0.21655 (17)	0.0220 (4)	
O6	0.3199 (4)	0.79731 (19)	0.08858 (15)	0.0194 (4)	
N1	1.1375 (5)	0.7234 (2)	0.4913 (2)	0.0222 (5)	
N2	0.9797 (5)	1.1792 (2)	0.1458 (2)	0.0191 (5)	
C1	0.8221 (5)	0.7323 (3)	0.3272 (2)	0.0172 (5)	
H1	0.7881	0.6315	0.3672	0.021*	
C2	1.0070 (5)	0.8080 (3)	0.3755 (2)	0.0175 (5)	
C3	1.0622 (5)	0.9551 (3)	0.3184 (2)	0.0164 (5)	
C4	0.9246 (5)	1.0237 (3)	0.2102 (2)	0.0172 (5)	
C5	0.7370 (5)	0.9523 (3)	0.1612 (2)	0.0171 (5)	
H5	0.6432	1.0033	0.0884	0.021*	
C6	0.6874 (5)	0.8061 (3)	0.2193 (2)	0.0168 (5)	
C7	0.4771 (5)	0.7304 (3)	0.1662 (2)	0.0168 (5)	
C8	0.2922 (6)	0.5029 (3)	0.1694 (3)	0.0244 (6)	
H8A	0.1036	0.5346	0.1860	0.037*	
H8B	0.3196	0.3969	0.2100	0.037*	
H8C	0.3221	0.5219	0.0807	0.037*	

### Atomic displacement parameters (Å^2^)

**Table:**

	*U*^11^	*U*^22^	*U*^33^	*U*^12^	*U*^13^	*U*^23^
Cl1	0.0217 (4)	0.0228 (4)	0.0222 (4)	−0.0051 (3)	−0.0061 (3)	−0.0083 (3)
O1	0.0396 (13)	0.0269 (11)	0.0254 (11)	−0.0063 (9)	−0.0043 (9)	−0.0013 (9)
O2	0.0279 (12)	0.0400 (13)	0.0325 (12)	−0.0039 (10)	−0.0142 (10)	−0.0035 (10)
O3	0.0229 (10)	0.0239 (10)	0.0253 (10)	−0.0084 (8)	0.0037 (8)	−0.0025 (8)
O4	0.0286 (11)	0.0177 (10)	0.0380 (12)	0.0006 (8)	−0.0006 (9)	−0.0093 (9)
O5	0.0251 (10)	0.0143 (9)	0.0260 (10)	−0.0062 (7)	−0.0078 (8)	−0.0035 (8)
O6	0.0213 (10)	0.0192 (9)	0.0165 (9)	−0.0052 (7)	−0.0055 (8)	−0.0017 (8)
N1	0.0279 (13)	0.0192 (12)	0.0189 (12)	−0.0012 (10)	−0.0085 (10)	−0.0037 (10)
N2	0.0213 (12)	0.0165 (11)	0.0196 (11)	−0.0053 (9)	−0.0058 (9)	−0.0042 (9)
C1	0.0209 (13)	0.0150 (12)	0.0145 (13)	−0.0007 (10)	−0.0014 (10)	−0.0020 (10)
C2	0.0191 (13)	0.0188 (13)	0.0148 (13)	0.0025 (10)	−0.0032 (10)	−0.0053 (10)
C3	0.0145 (12)	0.0191 (13)	0.0174 (13)	−0.0022 (10)	−0.0033 (10)	−0.0074 (11)
C4	0.0173 (13)	0.0136 (12)	0.0192 (13)	−0.0030 (10)	0.0014 (10)	−0.0022 (10)
C5	0.0159 (12)	0.0168 (12)	0.0179 (13)	−0.0018 (10)	−0.0025 (10)	−0.0035 (10)
C6	0.0167 (12)	0.0152 (12)	0.0177 (13)	−0.0015 (10)	−0.0013 (10)	−0.0034 (10)
C7	0.0166 (12)	0.0152 (12)	0.0167 (13)	−0.0039 (10)	0.0009 (11)	−0.0012 (10)
C8	0.0252 (15)	0.0154 (13)	0.0345 (16)	−0.0085 (11)	−0.0045 (12)	−0.0082 (12)

### Geometric parameters (Å, °)

**Table:**

Cl1—C3	1.725 (3)	C1—C6	1.393 (4)
O1—N1	1.242 (3)	C1—H1	0.9500
O2—N1	1.207 (3)	C2—C3	1.388 (4)
O3—N2	1.229 (3)	C3—C4	1.393 (4)
O4—N2	1.224 (3)	C4—C5	1.377 (3)
O5—C7	1.323 (3)	C5—C6	1.378 (3)
O5—C8	1.461 (3)	C5—H5	0.9500
O6—C7	1.202 (3)	C6—C7	1.507 (3)
N1—C2	1.477 (3)	C8—H8A	0.9800
N2—C4	1.473 (3)	C8—H8B	0.9800
C1—C2	1.390 (3)	C8—H8C	0.9800
			
C7—O5—C8	115.43 (19)	C5—C4—N2	118.5 (2)
O2—N1—O1	124.3 (2)	C3—C4—N2	118.8 (2)
O2—N1—C2	119.4 (2)	C4—C5—C6	119.1 (2)
O1—N1—C2	116.3 (2)	C4—C5—H5	120.4
O4—N2—O3	125.6 (2)	C6—C5—H5	120.4
O4—N2—C4	117.4 (2)	C5—C6—C1	120.3 (2)
O3—N2—C4	117.0 (2)	C5—C6—C7	118.5 (2)
C2—C1—C6	119.2 (2)	C1—C6—C7	121.1 (2)
C2—C1—H1	120.4	O6—C7—O5	125.7 (2)
C6—C1—H1	120.4	O6—C7—C6	122.5 (2)
C3—C2—C1	121.6 (2)	O5—C7—C6	111.8 (2)
C3—C2—N1	122.3 (2)	O5—C8—H8A	109.5
C1—C2—N1	116.1 (2)	O5—C8—H8B	109.5
C2—C3—C4	117.0 (2)	H8A—C8—H8B	109.5
C2—C3—Cl1	124.5 (2)	O5—C8—H8C	109.5
C4—C3—Cl1	118.43 (19)	H8A—C8—H8C	109.5
C5—C4—C3	122.7 (2)	H8B—C8—H8C	109.5
			
C6—C1—C2—C3	−0.5 (4)	O3—N2—C4—C5	−98.0 (3)
C6—C1—C2—N1	178.8 (2)	O4—N2—C4—C3	−97.7 (3)
O2—N1—C2—C3	−29.5 (4)	O3—N2—C4—C3	82.5 (3)
O1—N1—C2—C3	149.4 (3)	C3—C4—C5—C6	−1.7 (4)
O2—N1—C2—C1	151.2 (3)	N2—C4—C5—C6	178.8 (2)
O1—N1—C2—C1	−29.9 (3)	C4—C5—C6—C1	1.0 (4)
C1—C2—C3—C4	−0.1 (4)	C4—C5—C6—C7	178.7 (2)
N1—C2—C3—C4	−179.4 (2)	C2—C1—C6—C5	0.1 (4)
C1—C2—C3—Cl1	177.2 (2)	C2—C1—C6—C7	−177.6 (2)
N1—C2—C3—Cl1	−2.1 (4)	C8—O5—C7—O6	1.4 (4)
C2—C3—C4—C5	1.3 (4)	C8—O5—C7—C6	−179.4 (2)
Cl1—C3—C4—C5	−176.2 (2)	C5—C6—C7—O6	−12.6 (4)
C2—C3—C4—N2	−179.3 (2)	C1—C6—C7—O6	165.0 (2)
Cl1—C3—C4—N2	3.2 (3)	C5—C6—C7—O5	168.2 (2)
O4—N2—C4—C5	81.8 (3)	C1—C6—C7—O5	−14.2 (3)

### Hydrogen-bond geometry (Å, °)

**Table:**

*D*—H···*A*	*D*—H	H···*A*	*D*···*A*	*D*—H···*A*
C1—H1···O1^i^	0.95	2.51	3.329 (3)	145
C5—H5···O6^ii^	0.95	2.34	3.143 (3)	142

Symmetry codes: (i) −*x*+2, −*y*+1, −*z*+1; (ii) −*x*+1, −*y*+2, −*z*.
